# Severe pain-related adverse events of percutaneous dilatational tracheostomy performed by a neurointensivist compared with conventional surgical tracheostomy in neurocritically ill patients

**DOI:** 10.1186/s12883-020-01809-x

**Published:** 2020-06-03

**Authors:** Yong Oh Kim, Chi Ryang Chung, Chi-Min Park, Gee Young Suh, Jeong-Am Ryu

**Affiliations:** 1 Department of Critical Care Medicine, Samsung Medical Center, Sungkyunkwan University School of Medicine, Seoul, Republic of Korea; 2 Department of Medicine, Samsung Medical Center, Sungkyunkwan University School of Medicine, Seoul, Republic of Korea; 3 Department of Surgery, Samsung Medical Center, Sungkyunkwan University School of Medicine, Seoul, Republic of Korea; 4 Division of Pulmonary and Critical Care Medicine, Department of Medicine, Samsung Medical Center, Sungkyunkwan University School of Medicine, Seoul, Republic of Korea; 5 Department of Neurosurgery, Samsung Medical Center, Sungkyunkwan University School of Medicine, Seoul, Republic of Korea

**Keywords:** Percutaneous dilatational tracheostomy, Neurointensivist, Neurosurgery intensive care unit

## Abstract

**Background:**

We evaluated severe pain-related adverse events (SAE) during the percutaneous dilatational tracheostomy (PDT) procedure performed by a neurointensivist and compared the outcomes with that of conventional surgical tracheostomy in neurocritically ill patients.

**Methods:**

This was a retrospective and observational study of adult patients who were admitted to the neurosurgical intensive care unit between January 2014 and March 2018 and underwent tracheostomy. In this study, primary endpoints were incidence of SAE: cardiac arrest, arrhythmias, hypertension, hypotension, desaturation, bradypnea, or ventilatory distress. The secondary endpoint was procedure-induced complications.

**Results:**

A total of 156 patients underwent tracheostomy during the study. Elective surgery of brain tumors (34.0%) and intracranial hemorrhage (20.5%) were the most common reasons for admission. The most common reasons for tracheostomy were difficult ventilator weaning or prolonged intubation (42.9%) and sedative reduction (23.7%). Tachycardia (30.1%) and hypertension (30.1%) were the most common SAE. Incidence of SAE was more common in conventional tracheostomy compared to PDT (67.1% vs. 42.3%, *P* = 0.002). The total duration of SAE (19.8 ± 23.0 min vs. 3.4 ± 5.3 min, *P* < 0.001) and procedural time (42.2 ± 21.8 min vs. 17.7 ± 9.2 min, *P* < 0.001) were longer in conventional tracheostomy compared to PDT. Multivariable adjustment revealed that only PDT by a neurointensivist significantly reduced the incidence of SAE by one third (adjusted odds ratio [OR]: 0.36, 95% confidence interval [CI]: 0.187–0.691). In addition, PDT by a neurointensivist deceased the duration of SAE by 8.64 min (β: -8.64, 95% CI: − 15.070 – -2.205, *P* = 0.009) and prolonging the procedure time by every one minute significantly increased the duration of SAE by 6.38 min (β: 6.38, 95% CI: 0.166–0.470, *P* < 0.001). Procedure-induced complications were more common in conventional tracheostomy compared to PDT (23.5% vs. 11.3%, *P* = 0.047).

**Conclusions:**

This retrospective and exploratory study of our single-center limited cohort of tracheostomy patients revealed that decreased SAE may be associated with short procedural time during the PDT procedure performed by a neurointensivist. It is proposed that PDT by a neurointensivist may be safe and feasible in neurocritically ill patients.

## Background

Tracheostomy is generally performed in neurosurgical patients and may be performed more vigilantly in cases of brain injuries [1]. Several critical conditions should be considered such as intracranial hypertension, changed cerebral perfusion, and abnormal cerebral autoregulation if tracheostomy is performed in neurosurgical patients and neurocritically ill patients [1]. In such patients, procedural pain and anxiety may induce neurologically adverse events such as increased brain metabolism and intracranial hypertension [2–4]. Therefore, it is necessary to adequately control procedural pain and anxiety during the procedure in these patients.

Percutaneous dilatational tracheostomy (PDT) has been increasingly used because of easy placement and lower rates of clinically significant bleeding and wound infection [1, 5]. In addtion, a neurointensivist is specialist with a focus on the management of brain-injured patients with neurosurgical problems [6]. Therefore, PDT performed by a neurointensivist may have many theoretical advantages due to easy placement at the bedside, low risk of complications, and specialized management of brain-injured patients [1, 5]. In PDT performed by a neurointensivist, procedure induced severe pain-related adverse events (SAE) may be reduced. The purpose of this study was to investigate the SAE of PDT performed by a neurointensivist compared with conventional surgical tracheostomy in neurosurgical patients and neurocritcally ill patients.

## Methods

### Study population

**Fig. 1:**
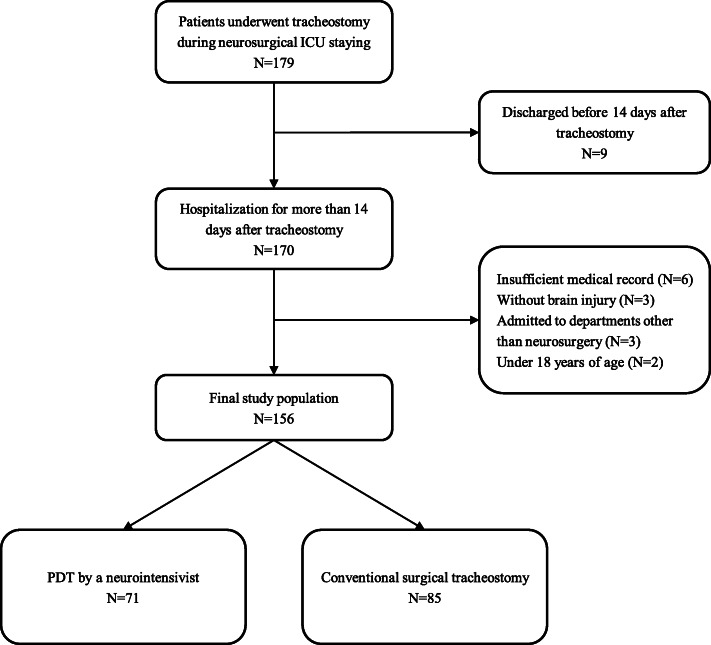
Study flow chart. ICU, intensive care unit; PDT, percutaneous dilatational tracheostomy

This was a retrospective, single-center, and observational study of adult patients admitted to the neurosurgical intensive care unit (ICU) at Samsung Medical Center between January 2014 and March 2018. This study was approved by the Institutional Review Board of Samsung Medical Center (SMC 2018–09-011). The requirement for informed consent was waived due to its retrospective nature. Clinical and laboratory data were collected by a trained study coordinator using a standardized case report form. We included adult patients admitted to the neurosurgical ICU who underwent tracheostomy during the study. The patient list was cross-referenced with the electronic order entry system and the electronic medical records to identify the patients who underwent tracheostomy during their ICU stay. Those hospitalized for more than 14 days after tracheostomy were selected. Of these patients, we excluded patients under 18 years of age, those without brain injury, and those with insufficient medical records. In addition, patients were excluded if they were admitted to departments other than neurosurgery (Fig. 1).

### Definitions and outcomes

We retrospectively reviewed all neurosurgical patients and neurocritcally ill patients who underwent tracheostomy and were admitted to the neurosurgical ICU during the study. All patients underwent a preliminary chart review to ensure that a T-cannula had been placed. The following baseline demographics were abstracted from the electronic medical chart including patient’s age and comorbidities, causes of ICU admission, severity scores on ICU admission, indication for tracheostomy, laboratory data on tracheostomy, vital signs monitored during the procedure, duration of the dwell time for the device, and causes of removal. As associated data of the tracheostomy procedure, we investigated the initial success of tracheostomy and procedure time. Procedure time was defined as the time from sterilization to the connection of the tracheostomy tube with the mechanical ventilator after successful tracheostomy [7, 8]. Primary endpoints were incidences of SAE: cardiac arrest, arrhythmias including tachycardia and bradycardia, hypertension, hypotension, desaturation, bradypnea, or ventilatory distress [9]. In this study, the incidence of SAE was mainly focused on brand new adverse events. SAE were retrospectively assessed by reviewing the medical records. SAE related to acute stress response were assessed based on physiological parameters (cardiac rhythm, heart rate, mean arterial pressure, respiratory rate, and oximetry) and measured continuously by the ICU monitor and recorded before and while the procedure by the bedside nurse on a sheet dedicated to the study. Tachycardia: heart rate ≥ 110 beats/minute (b/min) if < 100 b/min before the procedure; Bradycardia: heart rate ≤ 60 b/min if > 70 b/min before; Hypertension: mean arterial pressure ≥ 110 mmHg if < 100 mmHg before; Hypotension: mean arterial pressure ≤ 65 mmHg if > 70 mmHg before; Desaturation: oxygen saturation ≤ 90% if > 92% before; Bradypnea: respiratory rate ≤ 10 breaths/minutes (B/min) if > 10 B/min before; Ventilatory distress: severe ventilator asynchrony (non-stop coughing or impossible ventilation) in mechanically ventilated patients and/or tachypnea (respiratory rate ≥ 35 B/min if it was < 35 B/min) [9]. The secondary endpoint was defined as procedure-induced complications. We investigated complications at insertion or during maintenance such as insertional injury, bleeding or hematoma, fracture of the tracheal ring, cuff perforation, accidental decannulation, surgical conversion, hypoxemia, stomal ulcer, wound infection, and ventilator-associated pneumonia (VAP). As associated post-procedural bleeding complications, minor bleeding was defined as bleeding from the incision site that required dressing more than three times a day or epinephrine for local homeostasis. Major bleeding was defined as bleeding that required cauterization, surgical treatment, or additional blood transfusion [7, 8]. Arrhythmia developed during the procedure included bradycardia, sinus tachycardia, supraventricular tachycardia, arterial fibrillation, ventricular tachycardia, and ventricular fibrillation [7]. VAP was defined if patients received clinical diagnosis more than 48 h after tracheotomy, received antimicrobial therapy, and had a new radiographical infiltrate and two of three clinical criteria: purulent secretions, fever, and leukocytosis or leukopenia [1, 10]. VAP was also identified by cross-referencing with VAP monitoring of the Infection Prevention and Control Team at Samsung Medical Center. In this study, early and late-onset VAPs were defined as the occurrence of the events on four days or less and greater than four days after tracheostomy [11]. We defined initial success when the procedure was successfully performed at the first trial. The stroke-related early tracheostomy score (SETscore) was used to assess the need for tracheostomy and its external validation [12, 13]. In this study, a neurointensivist co-management was initiated in October 2014 [6]. A neurointensivist or neurosurgeon determined tracheostomy. Procedure of tracheostomy was performed in the same way as our previous study [5]. In PDT performed by a neurointensivist, patients received sedation and pain control with propofol or midazolam, intravenous analgesics with fentanyl and/or remifentanil, and topical analgesic with 1.5% lidocaine and 1:200,000 epinephrine [1, 5]. Especially, at least 30 min before the procedure, a continuously high dose infusion of propofol and bolus administration of osmotic agent were performed in patients with intracranial hypertension. Before the administration of the neuromuscular blockade, the goal of sedation was to achieve the Richmond Agitation Sedation Scale goal of negative 4 to 5 [5, 14, 15]. In tracheostomy performed by a neurointensivist and neurosurgeon, the first tracheostomy tube change was performed seven days after placement. However, it was performed three days after placement in conventional tracheostomy performed by an otolaryngologist [5].

### Statistical analyses

All data are presented as means ± standard deviations for continuous variables and numbers (percentages) for categorical variables. Data were compared using Student’s *t*-test for continuous variables and Chi-square test or Fisher’s exact test for categorical variables. Pearson’s correlation coefficient (*r*) was calculated to determine the correlation between the variables. To investigate independent and meaningful effects of clinically relevant variables, we considered a multivariable regression model, including variables such as age, sex, body mass index, Glasgow Coma Scale on tracheostomy, PDT by a neurointensivist, and use of sedative or analgesic. We used a multivariable linear model for the outcome of the duration of SAE and a multivariable logistic model for the outcome of the incidence of SAE. In the case of multivariable adjustment for the incidence of SAE, we paid extra attention to ensure adequate power of the analysis without overfitting and multicollinearity. All tests were two-sided and *P* values < 0.05 were considered as statistically significant. Data were analyzed using IBM SPSS statistics version 20 (IBM, Armonk, NY, USA).

## Results

### Baseline characteristics and procedural characteristics

A total of 156 patients (74 males; 47.4%) were analyzed in this study. Among these patients, 71 patients underwent PDT by a neurointensivist and 85 patients underwent conventional tracheostomy (61 by neurosurgeons and 24 by otolaryngologists) during the study period. Conventional tracheostomy was performed in eight (5.1%) patients in the operating room. The mean age of the patients was 59.6 ± 14.3 years. Hypertension (57.7%) and diabetes mellitus (28.8%) were the most common comorbidities among patients who underwent tracheostomy. Elective brain tumor surgery (34.0%) and intracranial hemorrhage (20.5%) were the most common reasons for ICU admission. There were no significant differences in age, gender, body mass index, comorbidities, and APACHE II score on ICU admission between the two groups except for the reason for admission and the Glasgow Coma Scale (Table 1). Difficult ventilator weaning or prolonged intubation (42.9%) and sedative reduction (23.7%) were the most common reasons for tracheostomy. Midazolam (70.3%) was most commonly used among sedatives and analgesics. There were no significant differences in laboratory results of coagulation on the day of tracheostomy between the two groups (Table 2).

**Table 1:** Baseline characteristics

	PDT performed by a neurointensivist (*n* = 71)	Conventional surgical method (*n* = 85)	*P* value
Age (yr) — mean ± SD	58.1 ± 15.5	60.8 ± 13.2	0.245
Gender, male — No. of patients (%)	35 (49.3)	39 (45.9)	0.671
BMI (kg/m^2^) — mean ± SD	23.7 ± 3.7	23.8 ± 4.0	0.815
Obese (BMI > 30 kg/m^2^) — No. of patients (%)	4 (5.6)	5 (5.9)	0.999
Comorbidities — No. of patients (%)			
Hypertension	38 (53.5)	52 (61.2)	0.335
Diabetes mellitus	23 (32.4)	22 (25.9)	0.371
Malignancy	23 (32.4)	13 (15.3)	0.012
Dyslipidemia	13 (18.3)	22 (25.9)	0.259
Chronic kidney disease	5 (7.0)	6 (7.1)	0.997
Coronary artery disease	4 (5.6)	5 (5.9)	0.999
Chronic liver disease	3 (4.2)	4 (4.7)	0.999
Reason for ICU admission — No. of patients (%)			0.012
Brain tumor	34 (47.9)	19 (22.4)	
Intracranial hemorrhage	10 (14.1)	22 (25.9)	
Traumatic brain injury	12 (16.9)	19 (22.4)	
Subarachnoid hemorrhage	8 (11.3)	20 (23.5)	
Cerebral infarction	2 (2.8)	4 (4.7)	
CNS infection	1 (1.4)	1 (1.2)	
Other	4 (5.6)	1 (1.2)	
GCS on ICU admission — mean ± SD	7.7 ± 4.0	6.2 ± 2.7	0.008
APACHE II score on ICU admission — mean ± SD	26.0 ± 5.1	27.4 ± 4.3	0.050

*PDT* Percutaneous dilatational tracheostomy, *SD* standard deviation, *BMI* body mass index, *TIA* transient ischemic attack, *ICU* intensive care unit, *CNS* central nerve system, *GCS* Glasgow Coma Scale, *APACHE* Acute Physiology and Chronic Health Evaluation

**Table 2:** Procedural characteristics at the time of tracheostomy

	PDT performed by a neurointensivist (*n* = 71)	Conventional surgical method (*n* = 85)	*P* value
Reason for tracheostomy — No. of patients (%)			0.853
Difficult ventilator weaning or prolonged intubation	33 (46.5)	34 (40.0)	
Reduction of sedative	15 (21.1)	22 (25.9)	
Airway protection or prevent the risk of aspiration	15 (21.1)	21 (24.7)	
Airway toilet	5 (7.0)	4 (4.7)	
Difficult airway	3 (4.2)	4 (4.7)	
Premedication — No. of patients (%)			
Midazolam	44 (62.0)	65 (76.5)	0.049
Cisatracurium	59 (83.1)	2 (2.4)	< 0.001
Fentanyl	55 (77.5)	2 (2.4)	< 0.001
Pethidine	15 (21.1)	29 (34.1)	0.106
Propofol	15 (21.1)	2 (2.4)	0.001
Vecuronium	11 (15.5)	2 (2.4)	0.008
Remifentanil	7 (9.9)	4 (4.7)	0.348
Succinylcholine	3 (4.2)	16 (18.8)	0.011
Other	5 (7.0)	8 (9.4)	0.808
Anticoagulation — No. of patients (%)	0 (0.0)	1 (1.2)	0.999
Use of antiplatelet agent — No. of patients (%)	5 (7.0)	1 (1.2)	0.093
GCS on procedure — mean ± SD	8.6 ± 3.7	8.3 ± 3.5	0.653
Use of renal replacement therapy — No. of patients (%)	3 (4.2)	3 (3.5)	0.999
Invasive ICP monitoring — No. of patients (%)	20 (28.2)	25 (29.4)	0.865
Duration of mechanical ventilation before tracheostomy (days) — mean ± SD	8.9 ± 5.8	8.2 ± 5.8	0.406
Inserted site — No. of patients (%)			0.045
1st tracheal membrane	16 (22.5)	35 (41.2)	
2nd tracheal membrane	39 (54.9)	34 (40.0)	
3rd tracheal membrane	16 (22.5)	16 (18.8)	
Internal diameter of T-cannula (mm) — No. of patients (%)			< 0.001
6.0	0 (0)	1 (1.2)	
7.0	1 (1.4)	22 (25.9)	
7.5	35 (49.3)	35 (41.2)	
8.0	33 (46.5)	24 (28.2)	
Adjustable flange	2 (2.8)	3 (3.5)	
Lab results on the day of tracheostomy — mean ± SD			
Platelet count (× 10^3^/μl)	252.2 ± 111.6	266.1 ± 107.0	0.440
PT (INR)	1.0 ± 0.2	1.0 ± 0.1	0.461
aPTT (sec)	38.9 ± 9.5	37.6 ± 6.4	0.330
Ventilator setting — mean ± SD			
FiO_2_ (%)	39.9 ± 18.6	36.0 ± 7.0	0.094
PEEP (cmH_2_O)	5.1 ± 0.8	4.9 ± 1.8	0.315
Vasopressor requirement — No. of patients (%)	11 (15.5)	13 (15.3)	0.973

*PDT* Percutaneous dilatational tracheostomy, *SD* standard deviation, *ICP* intracranial pressure, *INR* international normalized ratio, *aPTT* activated partial thromboplastin time, *FiO*_*2*_ Fraction of inspired oxygen, *PEEP* positive end-expiratory pressure

### Clinical outcomes

**Fig. 2:**
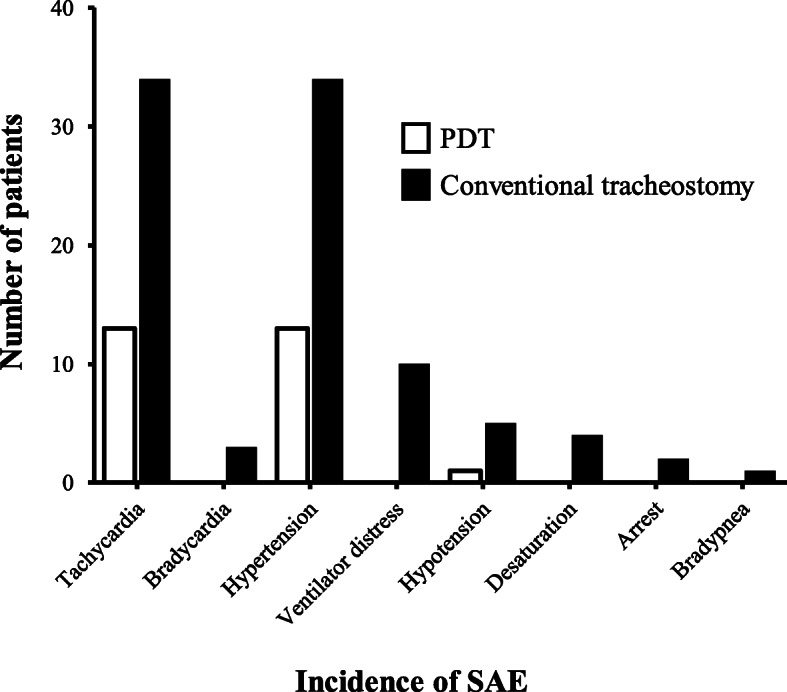
Incidence of severe pain-related adverse events (SAE). PDT, percutaneous dilatational tracheostomy

**Fig. 3:**
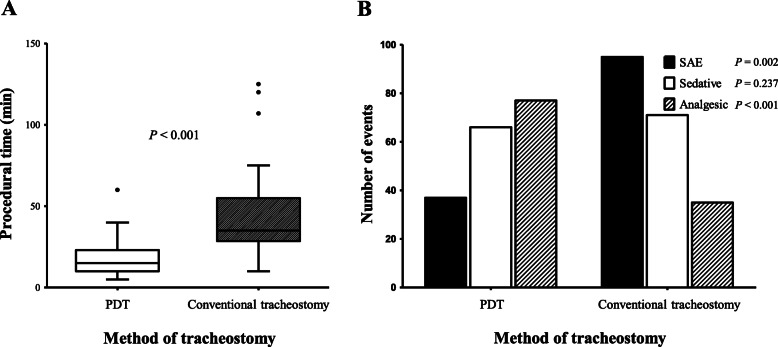
Procedural times (**a**) and severe pain-related adverse events (SAE) according to sedative and analgesic administration (**b**) between the two groups. PDT, percutaneous dilatational tracheostomy

**Fig. 4:**
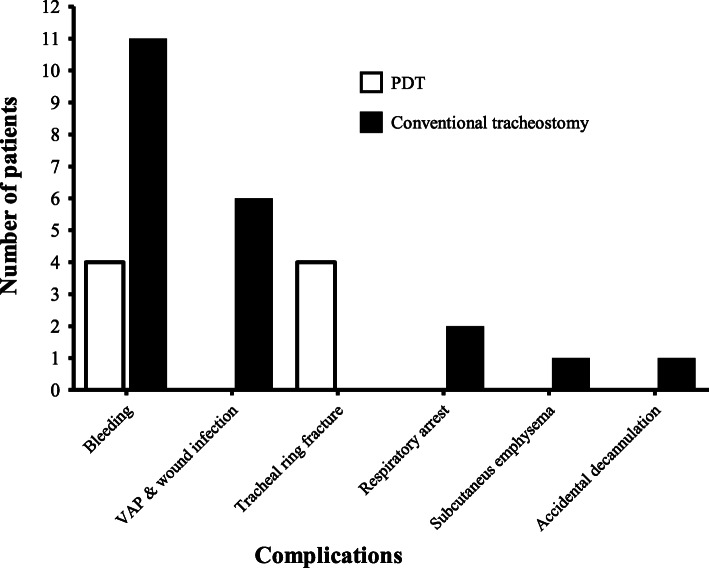
Procedure-induced complications. PDT, percutaneous dilatational tracheostomy; VAP, ventilator-associated pneumonia

Arrhythmia (30.4%) and hypertension (30.1%) were the most common events of SAE. Incidence of SAE was more common in patients who underwent conventional tracheostomy compared to patients who underwent PDT (67.1% vs. 42.3%, *P* = 0.002, Table 3, Fig. 2). The total duration of SAE was longer in patients who underwent conventional tracheostomy compared to patients who underwent PDT (19.8 ± 23.0 min vs. 3.4 ± 5.3 min, *P* < 0.001). In conventional tracheostomy, procedural time (42.2 ± 21.8 min vs. 17.7 ± 9.2 min, *P* < 0.001) was longer and analgesics were less used compared to PDT (38.8% vs. 84.5%, *P* < 0.001) (Table 2, Fig. 3). However, there was no difference in the use of sedatives between the two groups (81.2% vs. 73.2%, *P* = 0.237). Multivariable logistic regression analysis revealed that only PDT performed by a neurointensivist significantly reduced the incidence of SAE by one third (adjusted odds ratio [OR]: 0.36, 95% confidence interval [CI]: 0.187–0.691). Also, the duration of SAE was significantly different in cases of PDT performed by intensivists and surgeons. The SAE duration of PDT performed by neurointensivist (3.4 ± 5.3 min) was shortest compared with that of tracheostomy performed by an otolaryngologist (13.6 ± 16.7 min) and neurosurgeon (22.2 ± 24.7 min) (*P* < 0.001). Multivariable adjustment revealed that PDT by a neurointensivist exhibited a decrease in the duration of SAE by 8.64 min (β: -8.64, 95% CI: -15.070 – -2.205, *P* = 0.009) and prolonging the procedure time by every one minute significantly increased the duration of SAE by 6.38 min (β: 6.38, 95% CI: 0.166–0.470, *P* < 0.001). Procedure-induced complications were more common in patients who underwent conventional tracheostomy compared to patients who underwent PDT (23.5% vs. 11.3%, *P* = 0.047, Fig. 4). Although moderate or major bleeding occurred in six patients who underwent a conventional tracheostomy, only one patient had moderate bleeding in PDT. Especially, there were two respiratory arrests during conventional tracheostomy. Additionally, two early-onset VAPs and seven wound infections occurred in cases of conventional tracheostomy. However, there were no significant differences in mortalities and length of stay in the ICU and hospital between the two groups.

**Table 3:** Clinical outcomes

	PDT performed by a neurointensivist (*n* = 71)	Conventional surgical method (*n* = 85)	*P* value
Incidence of SAE — No. of patients (%)	30 (42.3)	57 (67.1)	0.002
Event of SAE — No. of events (%)			
Arrhythmia	13 (18.3)	36 (42.4)	0.001
Tachycardia	13 (18.3)	34 (40.0)	
Bradycardia	0 (0)	3 (3.5)	
Hypertension	13 (18.3)	34 (40.0)	0.003
Use of the antihypertensive drug during the procedure	7 (9.9)	11 (12.9)	0.728
Ventilator distress	0 (0)	10 (11.8)	0.008
Hypotension	1 (1.4)	5 (5.9)	0.303
Desaturation	0 (0)	4 (4.7)	0.179
Arrest	0 (0)	2 (2.4)	0.558
Bradypnea	0 (0)	1 (1.2)	0.999
Use of hyperosmolar agent during procedure	0 (0)	3 (3.5)	0.311
Duration of total SAE (min) — mean ± SD	3.4 ± 5.3	19.8 ± 23.0	< 0.001
Procedural data			
Initial success of tracheostomy — No. of patients (%)	71 (100)	81 (95.3)	0.179
Procedure time (min) — mean ± SD	17.7 ± 9.2	42.2 ± 21.8	< 0.001
Outcomes			
ICU mortality — No. of patients (%)	2 (2.8)	1 (1.2)	0.187
Hospital mortality — No. of patients (%)	6 (8.5)	9 (10.6)	0.651
Length of stay in ICU (days) — mean ± SD	17.35 ± 13.2	18.11 ± 18.4	0.773
Length of stay in hospital (days) — mean ± SD	78.74 ± 117.1	79.06 ± 136.2	0.988

*SAE* severe pain-related adverse events, *PDT* Percutaneous dilatational tracheostomy, *SD* standard deviation, *ICU* intensive care unit

**Fig. 5:**
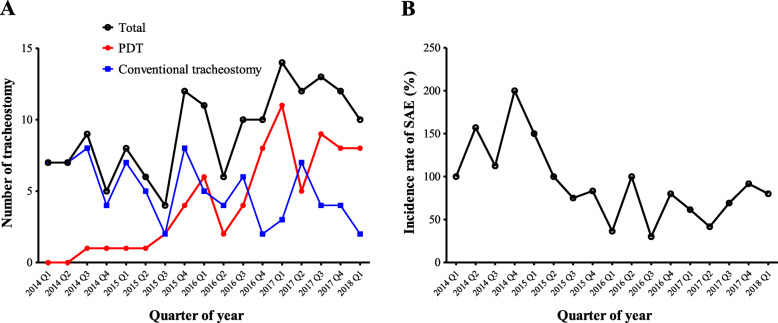
The number of total tracheostomy and PDT increased progressively compared to conventional tracheostomy (**a**). Especially, the total number of tracheostomy rapidly increased from the fourth quarter of 2015. Based on a simple correlation analysis, we found a linear trend with moderate to high correlation for the numbers of PDT, conventional surgical tracheostomy, and total tracheostomy (*r* = 0.915, *P* < 0.001, *r* = − 0.544, *P* = 0.024 and *r* = 0.667, *P* = 0.003, respectively). The overall incidence rate of SAE was gradually decreased (**b**). The incidence rate of SAE was calculated as the percentage of the number of SAE over the number of tracheostomies. We found a linear trend with high correlation for the incidence rate of SAE (*r* = − 0.655, *P* = 0.004)

In this study, the implementation of PDT was progressively increased compared to conventional tracheostomy (Fig. 5a). Especially, the total number of tracheostomy rapidly increased from the fourth quarter of 2015. There were no significant differences in the SETscore between the PDT group and the conventional tracheostomy group (15.9 ± 5.6 vs. 16.6 ± 5.3, *P* = 0.478). However, the SETscore before the fourth quarter of 2015 was significantly higher compared with that after the fourth quarter of 2015 (18.6 ± 5.0 vs. 14.6 ± 4.8, *P* < 0.001). Based on a simple correlation analysis, we found a linear trend with moderate to high correlation for the number of PDT, conventional surgical tracheostomy, and total tracheostomy (*r* = 0.915, *P* < 0.001, *r* = − 0.544, *P* = 0.024 and *r* = 0.667, *P* = 0.003, respectively). Besides, the overall incidence rate of SAE exhibited a gradual decrease (Fig. 5b). A linear trend with high correlation for the incidence rate of SAE was observed (*r* = − 0.655, *P* = 0.004).

Based on simple correlation analysis, the timing of tracheostomy showed a linear correlation with the length of ICU stay (*r* = 0.505, *P* < 0.001). However, the timing of tracheostomy was not significantly different between ICU survivors and non-survivors (9.3 ± 6.8 days vs. 13.2 ± 7.5 days, *P* = 0.328).

## Discussion

In this retrospective and exploratory study involving our single-center limited cohort of tracheostomy patients, we investigated the SAE of PDT performed by a neurointensivist at bedside compared with conventional surgical tracheostomy in neurosurgical patients. The major findings of this study are as follow: 1) After initiated neurointensivist co-management, the overall incidence rate of SAE was gradually decreased. Especially, incidence and duration of SAE were lower in patients who underwent PDT performed by a neurointensivist rather than in the case of patients who underwent conventional tracheostomy. Also, procedural time was longer in the conventional surgical tracheostomy group compared to the PDT group; 2) In multivariable analysis, the duration of SAE was associated with procedure time and operator. Incidence of SAE was only associated with PDT performed by a neurointensivist; 3) Procedure-induced complications were more common in patients who underwent conventional tracheostomy; and 4) In this study, implementation of total tracheostomy and PDT exhibited a progressive increase. However, the number of conventional surgical tracheostomy decreased gradually.

Pain is associated with acute stress response including changes in heart rate, blood pressure, respiratory rate, neuroendocrine secretion, and psychological distress such as agitation [9]. In neurocritically ill patients, including procedural pain, pain can increase cerebral metabolic demand which further increases intracranial pressure (ICP) [2–4]. Increased ICP can cause secondary brain ischemia due to decreased cerebral perfusion and blood flow, brain tissue hypoxia, metabolic crisis, and even result in herniation [16]. In addition, anxiety can increase cerebral metabolic demand which might further increase cerebral blood flow and ICP [2]. Sedation and analgesia are often required in the case of neurocritically ill patients with severe brain edema; both can also be used to manage ICP and cerebral perfusion pressure and attenuate stress response, increase endotracheal tube tolerance, reduce cerebral metabolic demand, prevent delirium, and decrease ventilator synchrony [16]. Therefore, appropriate levels of sedation and analgesia are necessary to control procedural pain and anxiety during tracheostomy in neurocritically ill patients.

In this study, neurointensivist co-management was initiated in October 2014. After initiated neurointensivist co-management, the overall incidence rate of SAE demonstrated a gradual decrease. There are several reasons behind the development of PDT as an appropriate skill for neurointensivists [1]. Neurointensivists are specialists with a focus on the management of patients with acute neurologic conditions including traumatic brain injury, stroke, status epilepticus, hypoxic-ischemic encephalopathy, and neuromuscular respiratory failure [6]. Frequency of tracheostomy is high among patients with neurocritically ill problems [17–22]. Neurointensivists need to be more vigilant in recognizing and managing subtle dangers that may occur during the procedure such as hypoventilation, head-down positioning, hypoxia, hypotension, and elevated ICP in case of brain-injured patients [20, 23, 24]. Herein, during PDT performance by a neurointensivist, continuous propofol infusion and bolus injection of hyperosmolar agents were initiated before the procedure in patients with intracranial hypertension. Therefore, a neurointensivist may have advantages of management and procedure in neurosurgical patients and neurocritically ill patients. Especially, decreased SAE might be associated with the initiation of neurointensivist co-management in this study.

In the current study, the number of total tracheostomy and PDT exhibited a progressive increase. Especially, the total number of tracheostomy cases rapidly increased from the fourth quarter of 2015. The SETscore before the fourth quarter of 2015 was significantly higher compared with that after the fourth quarter of 2015. Therefore, the indication for tracheostomy may be considered to be wider and easier compared with that of the past in patients with acute brain injury. A recent study reported an increase in tracheostomy utilization in the USA among patients with severe acute brain injury [25]. The increasing trend of tracheostomy has arisen from positive reasons such as easy weaning from the ventilator, facilitation of earlier discharge from intensive care, improved patient safety, etc. [25]. Also, tracheostomy and its timing have been hypothesized to be associated with clinical outcomes [26]. In this study, the timing of tracheostomy showed a linear correlation with the length of ICU stay. However, ICU mortality was not associated with the timing of tracheostomy.

This study has several limitations. First, it was a retrospective review of medical records. Especially, SAE were assessed by retrospectively reviewing of medical records. Vital signs and special events during the procedure were evaluated and recorded by the bedside nurses and not by an independent investigator. Therefore, there might be a bias according to each recorder. In addition, there existed a limitation to access the pain response in case a patient had abnormal vital signs before the procedure. Second, tracheostomy was determined by a neurointensivist or neurosurgeon, rather than by protocol-based procedure. Additionally, the non-randomized nature of the registry data may have resulted in selection bias. Third, the neurointensivist in our hospital is a neurologist trained to manage all aspects of critically ill patients in medical and surgical ICUs. The treatment tendency of this neurointensivist might have influenced clinical outcomes in neurosurgical patients. Finally, our study has limited statistical power due to the small sample size. Although numerous comparisons were made, there exists a possibility of the presence of false-positive findings in this study. However, the results still provide valuable insight. Consequently, prospective large-scale studies are needed to evaluate the usefulness of PDT by a neurointensivist for severe brain-injured patients to obtain evidence-based conclusions.

## Conclusions

In this retrospective and exploratory study of our single-center limited cohort of tracheostomy patients, we found that the incidence and duration of SAE were lower in PDT rather than conventional surgical tracheostomy. Decreased SAE may be associated with short procedural time and PDT performed by a neurointensivist. Therefore, it is proposed that PDT performed by a neurointensivist may be safe and feasible in neurocritically ill patients.

## Data Availability

Regarding data availability, our data are available on the Harvard Dataverse Network as recommended repositories of Springer Nature (10.7910/DVN/TH5DPC).

## References

[1] Seder DB, Lee K, Rahman C (2009). Safety and feasibility of percutaneous tracheostomy performed by neurointensivists. Neurocrit Care.

[2] Kukreti V, Mohseni-Bod H, Drake J (2014). Management of raised intracranial pressure in children with traumatic brain injury. J Pediatr Neurosci.

[3] Tan TK, Cheng MH, Sim EY (2015). Options for managing raised intracranial pressure. Proc Singapore Healthc.

[4] Ragland J, Lee K (2016). Critical care management and monitoring of intracranial pressure. J Neurocrit Care.

[5] Kwon J, Kim YO, Ryu J-A (2019). Safety and feasibility of percutaneous dilatational tracheostomy performed by a Neurointensivist compared with conventional surgical tracheostomy in neurosurgery intensive care unit. J Neurointensive Care.

[6] Ryu JA, Yang JH, Chung CR, Suh GY, Hong SC (2017). Impact of Neurointensivist co-management on the clinical outcomes of patients admitted to a neurosurgical intensive care unit. J Korean Med Sci.

[7] Lee D, Chung CR, Park SB, et al. Safety and Feasibility of Percutaneous Dilatational Tracheostomy Performed by Intensive Care Trainee. Korean J Crit Care Med. 2014;29(2):64–9.

[8] Lee DH, Jeong J-H (2018). Safety and feasibility of percutaneous dilatational tracheostomy in the Neurocritical care unit. J Neurocrit Care.

[9] de Jong A, Molinari N, de Lattre S (2013). Decreasing severe pain and serious adverse events while moving intensive care unit patients: a prospective interventional study (the NURSE-DO project). Crit Care.

[10] Michael SN, Donald EC (2005). Guidelines for the management of adults with hospital-acquired, ventilator-associated, and healthcare-associated pneumonia. Am J Respir Crit Care Med.

[11] Chastre J, Fagon JY (2002). Ventilator-associated pneumonia. Am J Respir Crit Care Med.

[12] Schonenberger S, Al-Suwaidan F, Kieser M, Uhlmann L, Bosel J (2016). The SETscore to predict tracheostomy need in cerebrovascular Neurocritical care patients. Neurocrit Care.

[13] Alsherbini K, Goyal N, Metter EJ (2019). Predictors for tracheostomy with external validation of the stroke-related early tracheostomy score (SETscore). Neurocrit Care.

[14] Sessler CN, Gosnell MS, Grap MJ (2002). The Richmond agitation-sedation scale: validity and reliability in adult intensive care unit patients. Am J Respir Crit Care Med.

[15] Giri PC, Bellinghausen Stewart A, Dinh VA, Chrissian AA, Nguyen HB (2015). Developing a percutaneous dilatational tracheostomy service by medical intensivists: experience at one academic institution. J Crit Care.

[16] Jeon SB, Koh Y, Choi HA, Lee K (2014). Critical care for patients with massive ischemic stroke. J Stroke.

[17] Bouderka MA, Fakhir B, Bouaggad A, Hmamouchi B, Hamoudi D, Harti A (2004). Early tracheostomy versus prolonged endotracheal intubation in severe head injury. J Trauma.

[18] Goettler CE, Fugo JR, Bard MR (2006). Predicting the need for early tracheostomy: a multifactorial analysis of 992 intubated trauma patients. J Trauma.

[19] Ahmed N, Kuo YH (2007). Early versus late tracheostomy in patients with severe traumatic head injury. Surg Infect.

[20] Stocchetti N, Parma A, Songa V, Colombo A, Lamperti M, Tognini L (2000). Early translaryngeal tracheostomy in patients with severe brain damage. Intensive Care Med.

[21] Rabinstein AA, Wijdicks EF (2004). Outcome of survivors of acute stroke who require prolonged ventilatory assistance and tracheostomy. Cerebrovasc Dis.

[22] Huttner HB, Kohrmann M, Berger C, Georgiadis D, Schwab S (2006). Predictive factors for tracheostomy in neurocritical care patients with spontaneous supratentorial hemorrhage. Cerebrovasc Dis.

[23] Stocchetti N, Parma A, Lamperti M, Songa V, Tognini L (2000). Neurophysiological consequences of three tracheostomy techniques: a randomized study in neurosurgical patients. J Neurosurg Anesthesiol.

[24] Milanchi S, Magner D, Wilson MT, Mirocha J, Margulies DR (2008). Percutaneous tracheostomy in neurosurgical patients with intracranial pressure monitoring is safe. J Trauma.

[25] Krishnamoorthy V, Hough CL, Vavilala MS (2019). Tracheostomy after severe acute brain injury: trends and variability in the USA. Neurocrit Care.

[26] Dasenbrock HH, Rudy RF, Gormley WB, Frerichs KU, Aziz-Sultan MA, Du R (2018). The timing of tracheostomy and outcomes after aneurysmal subarachnoid hemorrhage: a Nationwide inpatient sample analysis. Neurocrit Care.

